# RETRACTED: Atta et al. Application of Super-Amphiphilic Silica-Nanogel Composites for Fast Removal of Water Pollutants. *Molecules* 2016, *21*, 1392

**DOI:** 10.3390/molecules31081359

**Published:** 2026-04-21

**Authors:** Ayman M. Atta, Hamad A. Al-Lohedan, Ahmed M. Tawfik, Abdelrahman O. Ezzat

**Affiliations:** 1 Chemistry Department, College of Science, King Saud University, Riyadh 11541, Saudi Arabia; hlohedan@ksu.edu.sa (H.A.A.-L.); ao_ezzat@yahoo.com (A.O.E.); 2 College of Science, King Saud University, Riyadh 11541, Saudi Arabia; atawfik@ksu.edu.sa

The Journal retracts the article “Application of Super-Amphiphilic Silica-Nanogel Composites for Fast Removal of Water Pollutants” [1] cited above.

Following publication, concerns were brought to the attention of the Editorial Office regarding the integrity of a number of images presented in this publication [1].

In accordance with standard journal procedures, the Editorial Office and Editorial Board conducted an investigation that identified indications of inappropriate editing and duplication within Figure 1 presented in this article, as well as between Figures 1 and 2 of this article and Figures 1 and 4 of earlier publications [2,3,4], produced by a similar authorship group. Although the authors cooperated with the investigation, the explanations and supporting materials provided were not deemed sufficient by the Editorial Board to resolve the identified concerns. As a consequence, the Editorial Board has lost confidence in the reliability of the reported findings and has decided to retract this publication in accordance with MDPI’s retraction policy.

This retraction was approved by the Editor-in-Chief of the journal *Molecules.*

The authors have been informed of this retraction but did not provide a comment on this decision.
